# Longitudinal hematological dynamics during pregnancy and lactation in Thuringian Forest dairy goats under organic farming conditions

**DOI:** 10.14202/vetworld.2025.4184-4195

**Published:** 2025-12-31

**Authors:** Nina-li Brenner, Axel Wehrend, Henrik Werner Wagner, Abbas Farshad

**Affiliations:** Veterinary Clinic for Reproductive Medicine and Neonatology, Justus-Liebig-University of Giessen, 35392 Giessen, Germany

**Keywords:** blood parameters, dairy goats, hematology, lactation, organic farming, pregnancy, Thuringian Forest breed, veterinary diagnostics

## Abstract

**Background and Aim:**

Pregnancy and lactation place significant physiological demands on dairy goats, affecting red blood cell (RBC) indices, white blood cell (WBC) profiles, and platelet traits. Despite the diagnostic value of hematology (HA), there are no longitudinal, breed-specific reference values for Thuringian Forest goats. This study aimed to describe changes over time in differential blood counts and platelet indices in clinically healthy does kept under BIOLAND-certified organic management.

**Materials and Methods:**

A longitudinal study was conducted over one year using 25 clinically healthy Thuringian Forest does. Monthly blood samples were collected from 3 months prepartum through 12 months postpartum, resulting in 295 samples. Hematological analyses included RBC count, hematocrit (HCT), hemoglobin (HGB), mean corpuscular volume (MCV), mean corpuscular HGB (MCH), mean corpuscular HGB concentration (MCHC), RBC distribution width (RDW), and HGB distribution width (HDW). Platelet parameters, platelet count (PLT), mean platelet volume (MPV), and platelet distribution width (PDW), and differential WBC counts (neutrophils, lymphocytes, monocytes, eosinophils, and basophils) were measured using a validated automated analyzer. Repeated-measures analysis of variance evaluated the effects of reproductive stage, parity, milk yield, milk composition, and litter size.

**Results:**

Significant stage-dependent hematological changes were observed. RBC, HGB, and HCT decreased during late gestation and reached their lowest levels before birth, then increased gradually during lactation. MCV and MCH remained stable. PLT increased around parturition, MPV declined before birth and rose after, and PDW decreased steadily from late gestation through lactation. Neutrophils and total WBC counts increased toward parturition, while lymphocytes and monocytes rose during lactation. Eosinophils peaked at the start of lactation, and basophils declined after birth. Parity and milk yield significantly influenced certain RBC, platelet, and leukocyte parameters, whereas litter size showed no significant effect.

**Conclusion:**

Thuringian Forest goats show unique hematological changes during pregnancy and lactation, reflecting metabolic, hormonal, and immune adjustments related to reproduction and milk production. These breed-specific, stage-specific reference values improve clinical interpretation and diagnosis in dairy goat management. To our knowledge, this is the first longitudinal hematological study of this breed in organic farming conditions.

## INTRODUCTION

Goat farming is a vital part of global livestock production, supported by the adaptability, resilience, and productivity of major dairy breeds such as Anglo-Nubian, British Alpine, Toggenburg, and Saanen, which can produce up to 7.57 liters of milk per day under optimal conditions [1, 2]. Worldwide, goats contribute approximately 18.7 million tons of milk annually, nearly 2% of the global milk output, and the species’ population of 710 million animals across 570 recognized breeds reflects a level of genetic diversity similar to that of other major livestock species [1, 3, 4]. Although European breeds generally perform better in terms of body weight, litter size, and milk yield, many local or regional breeds remain poorly characterized due to the dominance of extensive management systems [1]. Goats are well-suited to a wide range of environmental conditions and production systems, making them valuable in both intensive and extensive farming contexts [5]. Their population growth, especially in developing regions, has outpaced that of cattle and sheep, highlighting their increasing importance in sustainable agriculture and food security strategies [6, 7].

Maintaining optimal herd health is especially crucial during physically demanding periods, such as pregnancy and lactation. Hematological analysis provides vital diagnostic insights by revealing changes in oxygen transport, immune response, and metabolic adaptation. Important indicators include red blood cell (RBC) count, hematocrit (HCT), hemoglobin concentration (HGB), and the white blood cell (WBC) profile [8–10]. However, many established reference values for goats are derived from sheep or cattle, highlighting the need for accurate, species-specific hematological reference intervals [8, 11–13]. Moreover, the accuracy of hematological measurements in goats depends on the analytical method used, as differences have been reported between automated hematology (HA) systems when measuring HGB and derived HGB variables in this species [14]. Hematological parameters are also affected by environmental and management factors, such as nutrition, housing, parity, season, and diet, which can modify physiological baselines [15–19].

Although reference values have been reported for several common dairy breeds [3, 5, 7, 12, 17–19], there is a notable lack of breed-specific hematological data for Thuringian Forest goats, especially during the critical reproductive stages of pregnancy and lactation.

Despite the growing global importance of dairy goats and the increasing reliance on HA for clinical assessment, there remains a significant lack of breed-specific hematological reference data for many regional goat populations. Existing reference intervals are mostly based on sheep, cattle, or a few widely studied dairy breeds such as Saanen, Alpine, and Damascus, which reduces diagnostic accuracy when applied to less-characterized breeds. Moreover, hematological parameters can vary greatly depending on reproductive stage, season, parity, management system, and nutritional environment, yet most available studies are cross-sectional, short-term, or limited to conventional farming systems. No longitudinal studies have focused on Thuringian Forest goats, a rare, regionally important dairy breed, despite their growing population and unique management under BIOLAND-certified organic systems. Critical physiological periods such as pregnancy and lactation, which involve major metabolic, endocrine, and immunological changes, remain undocumented for this breed. The lack of time-resolved, breed-specific, and system-specific hematological profiles presents a major diagnostic challenge for veterinarians, researchers, and organic dairy producers.

This study aimed to develop comprehensive, long-term hematological profiles for healthy Thuringian Forest dairy goats raised under BIOLAND-certified organic management, with a focus on reproductive stages during pregnancy and lactation. The goals were to describe how RBC indices, WBC counts, and platelet parameters change over time during the peripartum and lactation periods; to assess how parity, milk production, and milk composition affect key hematological indicators; and to establish physiologically relevant reference patterns for veterinary diagnostic use. By collecting high-frequency samples throughout an entire production year, this research aimed to differentiate normal adaptive hematological changes from potential disease-related alterations, providing the first breed- and system-specific dataset for Thuringian Forest goats and improving clinical evaluation accuracy in organic dairy herds.

## MATERIALS AND METHODS

### Ethical approval

All procedures involving animals in this study were carried out in strict accordance with the German Animal Welfare Act (Tierschutzgesetz, WAI) and were designed to minimize stress, pain, or discomfort to the animals. The experimental protocol, including all sampling procedures, animal handling steps, and welfare safeguards, was reviewed and approved by the Animal Welfare Office of Justus-Liebig-University Giessen (Institutional Animal Care and Use Committee equivalent). Ethical approval was granted under protocol number kTV 8-2017, dated May 30, 2017.

The study adhered to the European Union Directive 2010/63/EU on the protection of animals used for scientific purposes, the Animal Research: Reporting of *In Vivo* Experiments 2.0 guidelines for reporting in vivo research, and all relevant national and institutional standards governing the use of livestock in research. Only clinically healthy animals that met predefined welfare and health criteria (such as normal temperature, appetite, behavior, and no clinical infections) were included. Routine husbandry practices, including feeding, housing, and milking, remained unchanged for research, and blood sampling procedures were incorporated into the herd’s routine veterinary health monitoring to prevent additional stress.

All sampling was carried out by trained veterinarians or qualified personnel under the supervision of licensed veterinarians. Animals were handled gently with minimal physical restraint, and no invasive or painful procedures beyond routine jugular venipuncture were performed. No sedatives, anesthetics, or analgesics were needed due to the low invasiveness of the procedure. Goats were closely monitored before, during, and after each sampling to ensure full recovery and normal behavioral patterns.

The farm owner provided written informed consent before beginning data collection, authorizing the use of animals, clinical data, and production records solely for research and publication purposes. No animals were sold, rehomed, or euthanized as part of this study, and no experimental procedures impacted their ongoing production cycle.

Thus, the study followed the highest standards of animal welfare, ethical conduct, and regulatory compliance throughout all stages of design, sampling, and reporting.

### Study period and location

The study was conducted from February 2013 to October 2015. This long-term observational study was conducted on a BIOLAND-certified organic farm in the Rhein-Main area, which houses about 100 Thuringian Forest dairy goats, a breed that is still not well understood compared to more common dairy breeds such as Saanen, Alpine, and Damascus. The goats were kept in groups of 30–40 animals inside straw-bedded barns, with winter access to a covered concrete exercise yard and summer access to pasture between morning and evening milkings. Stocking density was kept consistent to reduce stress and social competition.

The region has a temperate climate with notable seasonal changes, with average temperatures from 2°C in winter to 28°C in summer, relative humidity between 65%–80%, and daylight hours shifting from about 8 h in December to over 16 h in June.

Routine herd monitoring ensured only clinically healthy goats were included. Eligibility criteria included normal temperature, appetite, behavior, and no visible infections. All goats were verified to be free of Caprine Arthritis-Encephalitis. Newly introduced animals underwent quarantine and veterinary checks as part of standard biosecurity procedures.

### Herd health management

Comprehensive health management included regular fecal egg counts several times a year, with selective deworming when parasite loads exceeded recommended thresholds. Vaccination protocols adhered to veterinary recommendations: pregnant does were immunized 4–6 weeks before kidding, and kids were vaccinated starting at 3 weeks old, with booster and yearly vaccinations administered afterward.

To prevent the enrollment of animals with subclinical disease, clinical examinations were supplemented with assessments of milk yield, udder health, and laboratory parameters. Goats showing signs of subclinical mastitis, metabolic imbalance, or any health issues were excluded. These steps ensured that only animals free of parasitic, infectious, or subclinical conditions participated in the study. This marks the first longitudinal, breed-specific study of hematological profiles in Thuringian Forest goats throughout pregnancy and lactation.

### Feeding and nutritional management

Goats were fed *ad libitum* farm-produced hay and received 500 g of crushed oats twice daily. Starting from the first day of milking, they also received 500 g of BIOLAND-certified dairy pellets daily. The pellet composition included wheat (6.5%), U-Ackerbeans (16%), wheat gluten feed (16%), wheat bran (13%), barley (9%), oats (6%), green meal (5%), wheat grits bran (4%), linseed cake (4%), and calcium carbonate (3.5%), fortified with vitamins A (12,500 I. E.), D_3_ (1,500 I. E.), and E (50 mg) per kg.

Pellet nutrient content included 18% crude protein, 7.5% crude fiber, 4% crude fat, 9% crude ash, 1.5% calcium, 0.5% phosphorus, and 0.4% sodium. Mineral lick stones containing macro- and microminerals, such as zinc (4,250 mg), manganese (2,500 mg), iron (2,000 mg), iodine (50 mg), selenium (45 mg), and cobalt (50 mg), were freely available. Water was supplied through automatic drinkers.

### Blood sampling procedure

A total of 25 goats, aged 2–3 years, were randomly selected from two barn groups, each consisting of 30–40 animals. Monthly blood samples were taken over 13 months, covering 15 physiological time points from 3 months before giving birth through late lactation. This frequent sampling enabled detailed, stage-specific monitoring of hematological changes and helped distinguish between physiological adaptations and pathological variations. All animals remained healthy throughout the study, and none dropped out. Any technical sample losses (such as clotting or instrument errors) were removed without replacing the data. Sampling was consistently conducted around 8:00 a.m., after morning milking, to reduce diurnal variation.

Blood was drawn from the external jugular vein using Neojekt® single-use cannulas (18G × 1½”) (Dispomed, Gelnhausen, Germany) with Monovette® collection systems (Sarstedt, Nürnbrecht, Germany). For each goat, four tubes were collected:

- one with 9 ml serum monovette (Sarstedt),
- one with 9 ml lithium-heparin monovette (16 IU/ml),
- one with 2.7 ml Ethylenediaminetetraacetate (ethylene diaminetetraacetic acid [EDTA], Sarstedt) monovette (1.6 mg/ml),
- one with 2.7 ml glucose monovette (Eickemeyer, Tuttlingen, Germany), (1.2 mg EDTA/ml and 1.0 mg fluoride/mL).

Samples were immediately cooled and protected from light. Serum, heparin, and glucose tubes were centrifuged at 2,150 × *g* for 10 min, and supernatants were transferred to tubes without additives. Serum and heparin samples were frozen at −20°C, while EDTA samples were stored cooled and analyzed within 24 h, ensuring parameter stability.

### Hematological analysis

EDTA blood samples were analyzed at the Central Laboratory, Faculty of Veterinary Medicine, Justus-Liebig-University Giessen, using the ADVIA 2120 hematology analyzer (Siemens Healthcare, Germany). This system has been validated for caprine and ovine blood, and previous studies confirm that EDTA-stabilized caprine blood maintains reliable hematological values, including differential counts, for up to 24 h with proper handling.

### Statistical analysis

Data were analyzed using a repeated-measures analysis of variance with a compound symmetry covariance structure to account for within-goat temporal correlations. Fixed effects included time point, parity (lactation number), milk yield, and litter size. Parity was explicitly incorporated into the model. Residual normality was evaluated through residual plots and Q-Q plots. Non-normal variables were transformed with logarithmic or square-root transformations. Normally distributed data were summarized as means, standard deviations, and minimum (xmin) and maximum (xmax) values. Transformed data were summarized with geometric means (x_g) and scatter factors. Tukey-adjusted post hoc comparisons controlled for type I error. Effect sizes (partial eta-squared, η²) and 95% confidence intervals were calculated for significant effects. Statistical significance was set at p < 0.05. Analyses were conducted using BMDP/Dynamic, Release 8.1 (DIXON, 1993, Los Angeles, CA, USA).

## RESULTS

### RBC parameters

**Figure 1:**
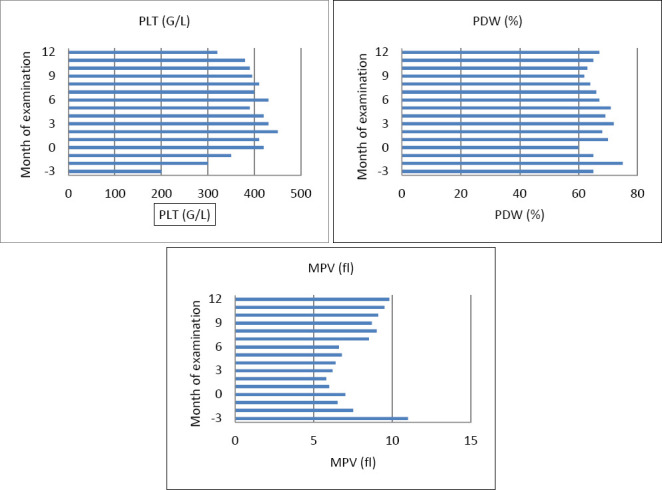
Platelet parameters in dairy goats measured monthly from three months pre-partum (-3) throughout the study period: (A) platelet count (PLT) [G/L] and (B) platelet distribution width (PDW) [%] are presented as mean ± standard deviation (SD); (C) mean platelet volume (MPV) [fL] is shown as geometric mean with scatter factor. Plots are shown as mean standard deviation (SD). The X-axis denotes the month of examination relative to the time of parturition (month 0). Critical physiological phases are indicated: late gestation (months –3 to –1), parturition (month 0), and early lactation (months +1 to +3), to aid interpretation of temporal changes.

Analysis of data from 295 blood samples (Table 1 and Supplementary 1) revealed significant changes in RBC (p = 0.0012), HCT (p < 0.0001), HGB (p = 0.0071), MCHC (p = 0.0017), RBC distribution width (RDW) (p < 0.0001), and HGB distribution width (HDW) (p < 0.0001) from 3 months prepartum to 1 month postpartum. Significant alterations were observed in RBC, HCT, HGB, MCHC, RDW, and HDW across this period, while MCV and MCH remained stable. RBC, HGB, and HCT declined during late gestation, reaching their lowest levels in the last month antepartum, then increased again during lactation. For example, HGB dropped from 8.2 ± 0.7 mmol/L at -3 months to 7.3 ± 0.3 mmol/L in late pregnancy, before rising to 9.1 ± 0.8 mmol/L at 11–12 months postpartum. No changes were observed in MCV and MCH (Figure 1). RBC, HGB, and HCT decreased toward birth, reaching their lowest values during the last month of pregnancy, then increased during lactation.

**Table 1:** The arithmetic means and standard deviations of the red blood cell (RBC, T/L), hematocrit (HCT, L/L), hemoglobin (HGB, mmol/L), mean corpuscular volume (MCV; fL), mean corpuscular hemoglobin (MCH, fmol), mean corpuscular hemoglobin concentration (MCHC, mmol/l]), RBC distribution width (RDW, %), and hemoglobin distribution width (HDW, mmol/l) in dairy goats over the study period, commencing three months pre partum (–3) with monthly sampling.

Phase	n	RBC	HCT	HGB	MCV	MCH	MCHC	RDW	HDW
Late gestation (3 to 1)	17	15.50 ± 1.05	0.307 ± 0.03	7.7 ± 0.5	19.8 ± 0.5	0.50 ± 0.02	25.3 ± 0.9	21.0 ± 1.2	1.85 ± 0.25
Parturition (0)	24	15.83 ± 1.43	0.314 ± 0.03	7.8 ± 0.7	19.9 ± 1.6	0.49 ± 0.03	24.8 ± 1.1	21.5 ± 1.1	2.02 ± 0.35
Early lactation (+1 to +3)	25	15.93 ± 1.32	0.287 ± 0.02	7.7 ± 0.5	18.1 ± 1.2	0.48 ± 0.02	26.7 ± 0.7	20.8 ± 1.0	1.61 ± 0.08
Mid-lactation (+4 to +6)	25	16.44 ± 1.66	0.293 ± 0.03	7.9 ± 0.7	17.9 ± 1.1	0.48 ± 0.03	27.1 ± 0.7	21.7 ± 0.9	1.61 ± 0.10
Late lactation (+7 to +12)	24	17.34 ± 1.74	0.310 ± 0.03	8.3 ± 0.8	17.8 ± 1.2	0.49 ± 0.03	27.7 ± 0.8	23.3 ± 1.3	1.53 ± 0.11

n = number of observations per phase. Supplementary Table S1 provides the full dataset with all parameters.

Significant correlations were observed between lactation number and RDW (p = 0.034) and HDW (p = 0.007); between milk yield and RDW (p = 0.026); between milk fat content and RBC (p = 0.0029), HGB (p = 0.0006), HKT (p = 0.011), HDW (p = 0.027); between milk protein content and RBC (p = 0.037); and between milk cell count and RBC (p = 0.041) and MCV (p = 0.049). Litter size did not influence the RBC count during lactation.

### Platelet dynamics (PLT, MPV, and PDW)

Moreover, PLT indices demonstrated significant time-dependent changes (Figure 1). PLT levels rose around parturition and early lactation, while MPV decreased until kidding and then increased again. PDW declined steadily from late gestation through lactation. Additionally, MPV increased with the number of lactations (+0.89 fL per lactation) but decreased with higher milk yield (–0.048 fL/kg). Associations with somatic cell count emphasize the impact of inflammatory status on platelet traits.

**Figure 2:**
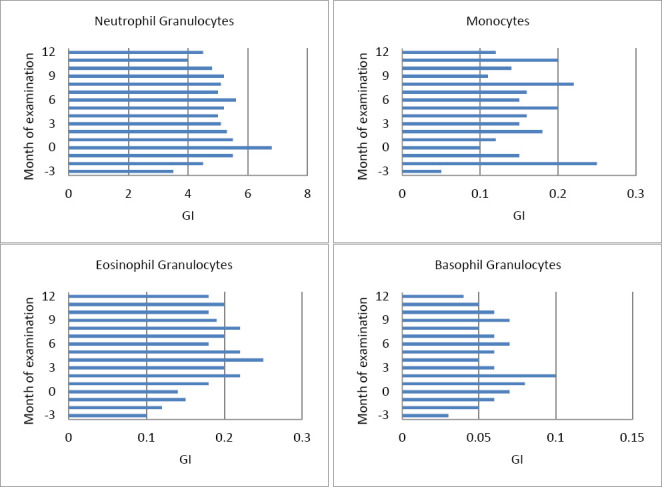
Geometric mean and scatter factor of (A) neutrophils (G/L), (B) monocytes (G/L), (C) eosinophils (G/L), and (D) basophils (G/L) in dairy goats, measured monthly from three months pre-partum (–3) throughout the study period. The X-axis represents the month of examination relative to the time of parturition (month 0). The critical physiological phases are indicated to aid interpretation: late gestation (months –3 to –1), parturition (month 0), and early lactation (months +1 to +3). The sample sizes (n) are shown above each bar. Error bars represent the variability across individuals.

Figure 2 shows the temporal changes in platelet count, PDW, and MPV in dairy goats. The PLT and PDW values were roughly normally distributed and are shown as arithmetic means with standard deviations. The MPV exhibited a right-skewed distribution and is reported as the geometric mean with a scatter factor.

Significant temporal changes in PLT (p < 0.0001) and MPV (p < 0.0001) were observed from 3 months prepartum to 1 month postpartum. PLT increased as parturition approached and during early lactation before stabilizing. MPV decreased significantly from 3 months prepartum to birth and increased during lactation. The PDW showed a steady decline from late gestation into the lactation period (p < 0.0001).

The MPV was positively associated with lactation number (p = 0.042), increasing by 0.89 fL for each additional lactation. Conversely, milk yield negatively affected MPV (p < 0.0001), decreasing it by 0.048 fL per kg. Cell counts significantly influenced all three parameters: MPV (p < 0.0006), PDW (p < 0.0013), and PLT (p = 0.0066). Specifically, an increase of 1 × 10³/mL in cell count raised MPV by 0.04 fL, PDW by 5.9%, and PLT by 58.7 G/L. There were no significant effects on milk fat or protein content, and no differences were observed in the mean PLT, MPV, or PDW between single and multiple births.

### WBC dynamics across reproductive stages

Leukocyte profiles also changed significantly throughout the peripartum period (Table 2 and Figure 2). Total leukocytes and neutrophils increased until parturition and then decreased afterward, while monocytes and lymphocytes increased during lactation. Eosinophils peaked at the start of lactation, whereas basophils decreased at 3 months postpartum. Parity had effects, with lymphocytes decreasing (–0.67 G/L per lactation) and monocytes increasing (×1.62), while milk yield positively influenced monocytes (+0.05 G/L per kg of milk).

**Table 2:** White blood cell count parameters (total leukocytes and lymphocytes) in goats, starting three months prepartum (-3). The smallest (x_min_), largest (x_max_) values, and arithmetic mean with standard deviation (Mean ± SD) are presented.

Phase	n	Total leukocyte count (G/L)	Lymphocytes [G/L]
Late gestation (3 to 1)	17	9.76 ± 2.15	3.87 ± 1.06
Parturition (0)	22	12.24 ± 3.05	4.47 ± 0.89
Early lactation (+1 to +3)	25	10.90 ± 2.26	4.52 ± 0.88
Mid-lactation (+4 to +6)	25	10.41 ± 2.14	4.42 ± 0.74
Late lactation (+7 to +12)	24	10.89 ± 2.54	4.54 ± 0.92

n = number of observations per phase, SD = Standard deviation. A full monthly dataset with minimum, maximum, and mean ± SD values is provided in Supplementary Table S2.

The total leukocyte and lymphocyte counts were normally distributed (Table 2 and Supplementary Information 2), while the neutrophil, eosinophil, basophil, and monocyte counts were skewed to the right (Figures 2A–D). As shown in Figure 1, the total leukocyte and lymphocyte counts were approximately normally distributed, whereas the neutrophil (A), monocyte (B), eosinophil (C), and basophil (D) counts exhibited right-skewed distributions.

Significant changes in leukocyte (p = 0.0051), neutrophil (A; p = 0.0006), eosinophil (C; p = 0.0004), basophil (D; p = 0.0005), and monocyte (B; p < 0.0001) counts were observed from 3 months prepartum to parturition. The lymphocyte count remained stable.

The neutrophil (A) and leukocyte counts increased toward birth and lactation onset, followed by a slight decline (p < 0.0001). Monocyte (B) and lymphocyte count modestly increased during lactation (both p < 0.0001). The eosinophils (C) increased at the start of lactation (p = 0.0073), while the basophils (D) decreased at 3 months postpartum.

The number of lactations affected lymphocyte (p = 0.0025) and monocyte (p = 0.001) counts, with each additional lactation decreasing lymphocyte counts by 0.67 G/L and increasing monocyte counts by 1.62 times. During lactation, lymphocyte levels declined by 0.4 G/L per lactation (p = 0.026). Milk yield was significantly positively correlated with monocytes (p = 0.0005), with every 1 kg increase in milk production raising monocyte counts by 0.05 G/L. Eosinophils (C) were significantly influenced by milk protein content (p = 0.033), increasing by 0.7 G/L for each 1% increase in protein. No significant differences in WBC parameters were observed between single and twin births.

## DISCUSSION

### Increasing importance of dairy goats and the need for longitudinal hematological research

The growing importance of dairy goats in hobby and commercial farming has increased the need for reliable veterinary diagnostics, yet long-term hematological studies in healthy goats, particularly the Thuringian Forest breed, remain limited. Hematological parameters are influenced by methodological, environmental, herd-specific, and physiological factors, including pregnancy [20–23]. This study examined changes in blood parameters over 1 year to gain insights into hematological variations during pregnancy and lactation. Such research is crucial for improving veterinary health monitoring and detecting HAs that could affect overall health and productivity [24, 25].

### Erythrocyte responses during pregnancy and lactation

The findings suggest that RBC parameters were mostly within the reference ranges, except for HDW values, which slightly exceeded the reported limits at certain points [26]. Pre-birth RBC, HGB, and HCT levels were low [27], but they increased during lactation [24]. Some studies show different trends, with RBC decreasing after birth [28]. Variations in methodology, age, environmental conditions, and herd-specific factors may explain these differences [29, 30]. Pregnancy increases plasma volume, leading to physiological hemodilution and temporarily lowering hematological values [31, 32]. Suppression of erythropoiesis by rising estrogen in late pregnancy may also contribute to changes in erythropoiesis [33, 34]. Lactation imposes significant metabolic demands and requires increased oxygen transport efficiency, which may explain the rise in RBC parameters after birth [24, 28].

### Endocrine regulation and hematological shifts

Endocrine regulation is likely to influence hematological changes during reproductive stages. Progesterone levels increase in pregnancy and may inhibit lymphocyte activity, helping maintain maternal immune tolerance. Estrogen, especially in late gestation, can decrease erythropoiesis, which aligns with lower prepartum RBC, HGB, and HCT levels. As estrogen and progesterone decline, coupled with increased prolactin and metabolic demand, erythropoietic recovery after birth may be stimulated.

Elevated cortisol levels during parturition and early lactation can cause neutrophilia and thrombocytosis, consistent with the observed neutrophil and platelet counts. Although hormonal levels were not directly measured, these mechanisms are in line with endocrine patterns reported in goats and other ruminants.

However, the lack of direct biochemical and hormonal profiling, such as cortisol, progesterone, calcium, and key metabolic biomarkers, restricts mechanistic interpretation. Including these parameters would allow for a more comprehensive understanding of endocrine–immune interactions and metabolic stress during pregnancy and lactation. Future research should incorporate hormonal and biochemical data to better interpret hematological fluctuations and support targeted diagnostic and management strategies.

### Platelet responses and periparturient stress

Thrombocyte counts remained within expected ranges, although fluctuations were observed across studies. Reference values for PDW and MPV in goats are absent, making assessments difficult. Increased thrombocyte numbers around birth and early lactation may indicate stress responses rather than regenerative activity [34, 35], as catecholamines boost platelet production during physiological stress [35].

### Leukocyte patterns and immune modulation

The WBC parameters were within the published reference ranges, supporting previous findings that goats typically have lymphocytic blood profiles [27, 36]. However, neutrophil counts often exceed lymphocyte numbers, likely due to handling-induced stress [26, 27]. Some breeds naturally show higher neutrophil proportions than lymphocytes [37], but further studies are needed to confirm this in Thuringian Forest goats.

Neutrophil dominance may also be linked to post-lambing inflammatory responses, including uterine involution. Leukocyte counts rose before lactation began and decreased afterward, aligning with studies that attribute this pattern to uterine involution [24, 25]. Some researchers have proposed that peripartum stress stimulates leukocyte production and release [28].

Lower lymphocyte counts during pregnancy compared to postpartum are consistent with physiological stress and immune modulation necessary to maintain fetal tolerance while preserving immune readiness [27, 38–41]. Monocyte counts declined during pregnancy and early lactation [42], although some studies have reported increases [28, 43]. These variations may be due to climate, milk yield, or environmental stressors [44, 45].

The basophil counts stayed within the expected ranges, but research during pregnancy and lactation is limited. This study showed a gradual increase in basophils into the third month of lactation, a pattern not previously documented [21, 25, 40], likely linked to immune recovery after pregnancy [46].

### Seasonal and environmental influences

Eosinophil counts peaked mid-study during summer pasture access, suggesting potential exposure to environmental allergens or parasites, although the counts remained within reference ranges. Seasonal fluctuations in eosinophils may relate to the growth of gastrointestinal nematodes during grazing months [44]. The increase indicates a possible immune response, despite the lack of clinical signs in this population.

### Linking HA to production traits

This study is among the first to examine correlations between hematological indices and milk yield and composition in goats. Our findings highlight the potential of hematological parameters to serve as functional biomarkers linking reproduction, health, and productivity, an approach rarely addressed in caprine research by integrating blood physiology with production outcomes.

### Comparison with existing literature and need for breed-specific data

Overall, pregnancy and lactation cause significant hematological changes in dairy goats. The longitudinal design, with no animal loss, ensured consistent sampling and reduced bias. The average values stayed within the published reference ranges, emphasizing the need to monitor blood profiles throughout reproductive cycles to distinguish normal physiological changes from pathological ones.

Further research on breed-specific differences, environmental factors, and nutrition could improve diagnostic accuracy and herd health management [47–49]. MCHC was affected by seasonal variation, while packed cell volume and PLT were significantly impacted; HGB, MCV, MCH, MPV, and platelet crit showed no notable seasonal changes [49].

### Consistency with recent studies (2020–2025)

In summary, hematological changes in Thuringian Forest goats during pregnancy and lactation align with recent studies on various breeds (2020–2025), showing that reproductive stage, parity, and environment affect erythrocyte indices, leukocyte profiles, and key biochemical markers [12, 50–62]. Late pregnancy and early lactation are linked to higher HGB and WBC levels due to increased oxygen needs and immune regulation [56, 57].

Seasonal influences, particularly temperature and humidity, affect immune stability and metabolism [17, 18, 58], and nutrition-based interventions can enhance biochemical resilience during climatic stress [59–61]. Parity influences serum glucose, total protein, and cholesterol levels, highlighting differences between primiparous and multiparous goats [16, 62]. Data from French Alpine and Arbia goats highlight mineral imbalances and stage-specific biochemical variability, supporting breed- and time-specific reference ranges [50, 51].

Emerging lipidomic and metabolomic evidence highlights transition-phase adaptations and emphasizes the need for personalized monitoring strategies in veterinary diagnostics [52, 59]. These insights reinforce the physiological patterns observed in this study and the importance of expanding controlled, breed-specific research to enhance herd health management.

### Translational value and future directions

In addition to providing breed- and stage-specific reference values, this study offers rare insights into platelet indices (PLT, MPV, and PDW) and leukocyte dynamics across reproductive stages. The surge in neutrophils around parturition and the increase in basophil counts during lactation highlight a periparturient immune adaptation rarely documented in goats, extending beyond typical RBC and WBC counts to provide a more complete picture of caprine physiology.

These stage- and breed-specific intervals have translational value for veterinary diagnostics, enable precise herd health monitoring, and provide a comparative framework for global standardization of goat HA. Additionally, the study establishes a foundation for future multidimensional physiological profiling that combines endocrine markers (progesterone, cortisol, and estrogen), biochemical parameters (glucose, calcium, and cholesterol), metabolomics, and production traits by providing this detailed longitudinal dataset. Such integration could support One Health approaches and welfare-focused research, positioning this work as pioneering baseline data for sustainable, breed-specific management strategies and advanced veterinary applications.

## CONCLUSION

This longitudinal study offers detailed insights into blood changes in healthy Thuringian Forest dairy goats throughout pregnancy and lactation, based on 295 blood samples collected over a year. The research showed clear stage-related shifts in RBC, HGB, and HCT levels: they decreased during late gestation due to hemodilution and hormonal regulation, then increased during lactation in response to higher oxygen demands. Platelet indices showed distinct patterns around parturition: PLT rose near birth and MPV decreased before and increased after birth, while PDW steadily decreased. Leukocyte patterns signified coordinated immune adjustments, with prepartum increases in total leukocytes and neutrophils, postpartum rises in lymphocytes and monocytes, eosinophil peaks at the start of lactation, and decreasing basophils later in lactation. Links between hematological traits, parity, milk production, and composition highlight the interconnectedness of metabolic, productive, and immune functions. Overall, these results establish breed- and stage-specific reference trends that aid in the more accurate interpretation of blood tests in veterinary practice.

The practical importance of these results lies in their use for clinical decision-making, especially for distinguishing between normal reproductive physiology and early signs of disease, metabolic imbalance, or inflammatory stress. These reference values are particularly useful for organic goat farms, where preventive health care depends mainly on physiological monitoring rather than drug treatment. The strengths of this study include its longitudinal design, high sampling frequency, careful selection of healthy animals, consistent management conditions, and detailed integration of hematological, reproductive, and production data. However, its limitations include being conducted at a single farm, a small sample size, and the lack of additional biochemical and hormonal markers, which limit understanding of some hematological changes. Moreover, the specific conditions of BIOLAND-certified management may make it difficult to apply these findings to intensive or semi-intensive systems.

Future research should incorporate endocrine profiles, metabolic biomarkers, mineral status, and advanced omics techniques to clarify the mechanistic pathways responsible for hematological adaptation during reproduction. Studies involving multiple herds and breeds would further enhance external validity and promote global standardization of goat HA.

In conclusion, this study provides the first detailed longitudinal hematological baseline for Thuringian Forest goats, highlighting distinct periparturient physiological adjustments and underscoring the importance of dynamic monitoring for herd health management. These findings improve breed-specific diagnostic interpretation, support reproductive and welfare management, and establish a crucial foundation for future multidimensional physiological research in dairy goats.

## DATA AVAILABILITY

The supplementary data can be made available from the corresponding author upon request.

## AUTHORS’ CONTRIBUTIONS

NB: Sample collection and data recording, analysis, and interpretation. NB, AW, HW, and AF: Formal analysis, data curation, and writing – review & editing. AF: Conceptualization. All authors have read and agreed to the publication of the final version of the manuscript.
